# Evaluation of Adiposity and Cognitive Function in Adults

**DOI:** 10.1001/jamanetworkopen.2021.46324

**Published:** 2022-02-01

**Authors:** Sonia S. Anand, Matthias G. Friedrich, Douglas S. Lee, Phillip Awadalla, J. P. Després, Dipika Desai, Russell J. de Souza, Trevor Dummer, Grace Parraga, Eric Larose, Scott A. Lear, Koon K. Teo, Paul Poirier, Karleen M. Schulze, Dorota Szczesniak, Jean-Claude Tardif, Jennifer Vena, Katarzyna Zatonska, Salim Yusuf, Eric E. Smith

**Affiliations:** 1 Population Health Research Institute, McMaster University and Hamilton Health Sciences, Hamilton, Ontario, Canada; 2 Department of Medicine and Epidemiology, McMaster University, Hamilton, Ontario, Canada; 3 Department of Cardiology and Diagnostic Radiology, McGill University, Montreal, Quebec, Canada; 4 Programming and Biostatistics, Institute for Clinical Evaluative Sciences, Toronto, Ontario, Canada; 5 Division of Cardiology, Peter Munk Cardiac Centre and University Health Network, University of Toronto, Toronto, Ontario, Canada; 6 Department of Molecular Genetics, Ontario Institute for Cancer Research, University of Toronto, Toronto, Ontario, Canada; 7 Department of Kinesiology, University of Laval, Quebec City, Quebec, Canada; 8 Department of Health Research Methods, Evidence, and Impact, McMaster University, Hamilton, Ontario, Canada; 9 Department of Epidemiology, Biostatistics, and Public Health Practice, School of Population and Public Health, University of British Columbia, Vancouver, British Columbia, Canada; 10 BC Cancer Agency, Vancouver, British Columbia, Canada; 11 Department of Medical Biophysics, Robarts Research Institute, Western University, London, Ontario, Canada; 12 Department of Medicine, University of Laval, Quebec City, Quebec, Canada; 13 Faculty of Health Sciences, Simon Fraser University, Burnaby, British Columbia, Canada; 14 Faculté de Pharmacie, Institut Universitaire de Cardiologie et de Pneumologie de Québec, Quebec City, Quebec, Canada; 15 Department of Psychiatry, Wroclaw Medical University, Wrocław, Poland; 16 Montreal Heart Institute, Université de Montréal, Montreal, Quebec, Canada; 17 Cancer Research & Analytics, Cancer Care Alberta, Alberta Health Services, Edmonton, Alberta, Canada; 18 Department of Social Medicine, Wroclaw Medical University, Wrocław, Poland; 19 Department of Clinical Neurosciences and Hotchkiss Brain Institute, Cumming School of Medicine, Calgary, Alberta, Canada; 20 University of Calgary, Calgary, Alberta, Canada; 21 Department of Clinical Neurosciences, University of Calgary, Calgary, Alberta, Ontario, Canada

## Abstract

**Importance:**

Excess adipose tissue increases other cardiovascular risk factors, which may be associated with vascular brain injury and cognitive impairment. However, the extent to which the amount and distribution of adipose tissue may be associated with lower cognitive scores, independent of its association with cardiovascular risk factors, is not well characterized.

**Objective:**

To investigate the association of adiposity on vascular brain injury and cognitive scores.

**Design, Setting, and Participants:**

A total of 9189 participants from the Canadian Alliance for Healthy Hearts and Minds (CAHHM) and the Prospective Urban Rural Epidemiological–Mind (PURE-MIND) cohort studies were included in this cross-sectional analysis. Of these adults, 9166 underwent bioelectrical impedance analysis to assess body fat (BF) percentage, and 6773 underwent magnetic resonance imaging to assess vascular brain injury and measure visceral adipose tissue (VAT) volume. Participants from CAHHM were recruited from January 1, 2014, to December 31, 2018, and PURE-MIND participants were recruited from January 1, 2010, to December 31, 2018. Both CAHHM and PURE-MIND comprise multisite, population-based cohorts. Participants from CAHHM are from Canada, and PURE-MIND participants are from Canada or Poland. Data analysis was performed from May 3 to November 24, 2021.

**Exposures:**

The percentage of BF and VAT were modeled as sex-specific quartiles. Vascular brain injury was defined as high white matter hyperintensities or silent brain infarction. Multivariable mixed models were used to examine factors associated with reduced cognitive scores.

**Main Outcomes and Measures:**

Cognitive function was assessed using the Digital Symbol Substitution Test (DSST; scores range from 0 to 133, with lower scores indicating lower cognitive function) and Montreal Cognitive Assessment (scores range from 0 to 30, with a score of ≥26 denoting normal cognitive function). Reduced cognition was defined as a DSST score less than 1 SD below the mean. Cardiovascular risk was assessed using the INTERHEART Risk Score (IHRS; scores range from 0 to 48; low risk is defined as a score of 0 to 9, moderate risk as 10 to 16, and high risk as 17 or higher).

**Results:**

A total of 9189 adults (mean [SD] age, 57.8 [8.8] years; 5179 [56.4%] women; and 1013 [11.0%] East and Southeast Asian; 295 [3.2%] South Asian; 7702 [83.8%] White European; and 179 [1.9%] other, including Black, Indigenous, mixed, and unknown ethnicity) participated in the study. Visceral adipose tissue was highly correlated with body adiposity measured by BF percentage (*r* = 0.76 in women; *r* = 0.70 in men). Cardiovascular risk factors increased with increasing BF percentage with the fourth quartile IHRS at 13.8 (95% CI, 13.5-14.0; *P* < .001 for trend) and with VAT with the fourth quartile IHRS at 13.3 (95% CI, 13.0-13.5; *P* < .001 for trend). Vascular brain injury increased with increasing BF percentage with the fourth quartile value at 8.6% (95% CI, 7.5%-9.8%; *P* = .007 for trend) and with increasing VAT with fourth quartile value at 7.2% (95% CI, 6.0-8.4; *P* = .05 for trend). Cognitive scores were lower with increasing BF percentage with the fourth quartile score of 70.9 (95% CI, 70.4-71.5; *P* < .001 for trend) and for VAT with the fourth quartile score of 72.8 (95% CI, 72.1-73.4; *P* < .001 for trend). For every 1-SD increase in BF percentage (9.2%) or VAT (36 mL), the DSST score was lower by 0.8 points (95% CI, 0.4-1.1; *P* < .001) for BF percentage and lower by 0.8 points (95% CI, 0.4-1.2; *P* < .001) for VAT, adjusted for cardiovascular risk factors and vascular brain injury. The population attributable risk for reduced DSST score for higher BF percentage was 20.5% (95% CI, 7.0%-33.2%) and for VAT was 19.6% (95% CI, 2.0%-36.0%). Higher BF percentage and VAT were not associated with Montreal Cognitive Assessment scores.

**Conclusions and Relevance:**

In this cross-sectional study, generalized and visceral adiposity were associated with reduced cognitive scores, after adjustment for cardiovascular risk factors, educational level, and vascular brain injury. These results suggest that strategies to prevent or reduce adiposity may preserve cognitive function.

## Key Points

**Question:**

To what extent are the amount and distribution of adipose tissue associated with cognitive scores, independent of their association with cardiovascular risk factors?

**Findings:**

In this cross-sectional analysis of 9189 adults between 30 and 75 years of age who were free of cardiovascular disease from the Canadian Alliance for Healthy Hearts and Minds (CAHHM) and the Prospective Urban Rural Epidemiological–Mind (PURE-MIND) cohort studies, higher body fat percentage and visceral adipose tissue were associated with more cardiovascular risk factors, vascular brain injuries, and lower cognitive scores.

**Meaning:**

The results of this study suggest that generalized and visceral adipose tissue are associated with reduced cognitive scores, after adjustment for cardiovascular risk factors and vascular brain injury.

## Teaser

This cross-sectional study of adults without cardiovascular disease assesses whether the amount and distribution of adipose tissue are associated with cognitive scores.

## Introduction

Generalized adiposity is associated with higher levels of cardiovascular risk factors, including diabetes, hypertension, elevated cholesterol levels, and cardiovascular disease (CVD).^1,2^ Total body adiposity is also associated with increased circulating markers of inflammation, which may contribute to increased CVD risk, independent of other cardiovascular risk factors.^3,4,5^ Central adiposity, measured by abdominal waist circumference, is a surrogate measure of the abdominal distribution of adipose tissue and is more strongly associated with myocardial infarction than is body mass index.^2^ Magnetic resonance imaging (MRI) can detect visceral adipose tissue (VAT) volume, which reflects the adipose tissue stored within the abdominal cavity. The metabolic properties of VAT are thought to be distinct from subcutaneous adipose tissue because its presence is strongly correlated with cardiovascular risk factors, such as elevated glucose levels, blood pressure, and atherogenic lipoprotein levels.^5,6^ Excess VAT is considered to be a consequence of the relative inability of subcutaneous adipose tissue to expand through hyperplasia when facing an energy surplus, leading to a lipid spillover and ectopic fat deposition in normally lean tissues (eg, heart, liver, skeletal muscle, pancreas, and kidney).^7^ Furthermore, VAT is believed to be the source of increased circulating inflammatory proteins, which are associated with increased CVD.^7^

The association between total body and central adiposity and cognitive function is uncertain. The surrogate measures of VAT commonly used in epidemiologic studies, including the waist circumference or waist to hip ratio (WHR), have been examined in association with cognitive function. In general, cross-sectional evaluations^8,9,10^ have suggested that increased central adiposity is associated with reduced cognitive scores, and a prospective evaluation^11^ found that increased central adiposity in African Americans was associated with a faster rate of cognitive decline during a 5-year follow-up. More recently, cross-sectional investigations using radiologic evaluations of VAT by computed tomography or MRI have had mixed results with respect to cognitive function, with some finding an inverse association between increased VAT and cognitive function^12^ and others finding a protective effect of VAT on cognitive function.^13^ We investigated the association between the amount and distribution of adipose tissue and cognitive function scores after adjustment for other cardiovascular risk factors and MRI-detected vascular brain injury among men and women free of clinical CVD.

## Methods

The enrollment criteria for the Canadian Alliance for Healthy Hearts and Healthy Minds (CAHHM)^14^ and Prospective Urban Rural Epidemiological–Mind (PURE-MIND) participants from Canada and Poland^15^ were similar and included adults between the ages of 30 and 69 years for CAHHM and 40 and 75 years for PURE-MIND. The CAHHM participants were recruited from January 1, 2014, to December 31, 2018, and PURE-MIND participants were recruited from January 1, 2010, to December 31, 2018. Data analysis was performed from May 3 to November 24, 2021. Research ethics board approval was obtained from the Hamilton Integrated Research Ethics Board, and all participants provided written informed consent. All data were deidentified. This cross-sectional study is reported according to Strengthening the Reporting of Observational Studies in Epidemiology (STROBE) reporting guideline (eTable 1 in the Supplement).

Adults with clinical CVD, defined as a history of stroke, coronary artery disease, heart failure, or other heart disease, were excluded. A total of 9189 CAHMM and PURE-MIND participants were included; 9166 had body fat (BF) percentage measured by bioelectrical impedance analysis and 6773 underwent MRI of the abdomen to measure VAT volume (eFigure in the Supplement). All underwent MRI of the brain to measure vascular brain injury, including silent brain infarctions and high white matter hyperintensities (HWMHs).^16^ Cardiovascular risk factors were measured using health and lifestyle questions and physical measures, and cognitive assessment was measured by the Digital Symbol Substitution Test (DSST) and the Montreal Cognitive Assessment (MoCA).^16^

### Key Measures

#### Total Body Adiposity

The bioelectrical impedance analysis measure of total body adiposity was determined using the Tanita Ironman, Innerscale BC-554, which provides a measure of total BF percentage.

#### Cardiovascular Risk Factors

The non–laboratory-based INTERHEART Risk Score (IHRS) is a validated score that is incrementally associated with silent brain infarction^17^ and quantified cardiovascular risk factor burden. The IHRS includes age, sex, smoking status, diabetes, high blood pressure, family history of myocardial infarction, WHR, home or work social stress, depression, dietary habits, and physical activity.^17,18^ The IHRS scores range from 0 to 48; low risk is defined as a score of 0 to 9, moderate risk as 10 to 16, and high risk as 17 or higher. In a subset of participants who provided blood samples (n = 4492), apolipoprotein B and A1 were measured. For educational level, participants were classified as having high school (or less), trade or technical training, and any college and/or a university earned certificate, bachelor’s degree, or a graduate degree.

### Cognitive Assessment

The DSST is a 2-minute test that requires participants to match symbols with numbers according to a code^19^ and assesses visual-motor speed and coordination, capacity for learning, attention, concentration, and short-term memory. Scores range from 0 to 133, with lower scores indicating worse performance. Reduced cognitive function is defined as those with a DSST score less than 1 SD (15.7) below the mean (74.0) based on the CAHHM participants’ data. The MoCA is a 10- to 15-minute, global, cognitive, interviewer-administered screening test that evaluates delayed recall, verbal fluency, visuospatial skills, executive functions, calculation, abstraction, language, orientation, attention, and concentration. Scores range from 0 to 30, and a score of 26 or higher denotes normal cognitive function.^20^

#### Magnetic Resonance Imaging

Details of the CAHHM and PURE-MIND MRI protocols have been previously published.^14,15^ Briefly, participants underwent a short noncontrast enhanced scan using a 1.5-T or 3-T magnet of the brain and abdomen.

#### Brain

Key brain injury measures included HWMH burden and silent brain infarctions. The white matter hyperintensity was rated on the Fazekas score, a visual rating scale validated to correlate with volumetric measurements. High was defined as a Fazekas score of 4 or higher (summing the periventricular and subcortical grades), which indicates beginning confluent or confluent white matter hyperintensity.^16^ Individuals with no prior history of stroke and with MRI evidence of 1 or more areas of brain infarction were classified as having a silent brain infarct. A small subcortical silent brain infarct, with an axial diameter of 15 mm or less, was classified as a lacunar silent brain infarct. A cortical silent brain infarct of any size or a subcortical silent brain infarct greater than 15 mm was classified as a nonlacunar silent brain infarct. An MRI-detected vascular brain injury was defined as the presence of HWMHs or silent brain infarct, including lacunar and nonlacunar infarctions.

#### Visceral Adipose Tissue

Abdominal VAT was determined by sequences heavily weighted for T1, providing a bright signal for fat. Visceral adipose tissue is derived from the T1-weighted turbo spin echo axial sequence through L4-L5. Visceral adipose tissue volumes were analyzed and reported by the core laboratory, and sex-stratified quartiles were derived for analysis.

### Statistical Analysis

The key adiposity exposures, percentage of BF and VAT, were modeled both as continuous measures and with sex-specific quartiles using a complete case analysis; all missing values were assumed to be missing at random and no imputations were performed. Comparisons of risk factors and cognitive function scores across adiposity quartiles were adjusted for age, sex, and ethnicity, and cognitive scores were further adjusted for completed education. *P* value for trend was calculated using linear contrasts. For each adiposity measure, linear mixed models were fitted to determine the joint effects of adiposity, age, sex, height, ethnicity, educational level, MRI-detected vascular brain injury variables, and the IHRS on the mean change in DSST score and separately for the MoCA. A random effect for center was included in the model, and the covariance matrix was specified to be unstructured. Height was included to account for differences in body size. To consider the sensitivity of the final multivariable models, they were repeated in a healthy cohort (removing those with a history of diabetes or hypertension) and in a cohort in which WHR was removed from the IHRS because WHR is a measure of adiposity and thus associated with percentage of BF and VAT. Cognitive aging is estimated from the linear regression model by dividing the β coefficient of the effect of adiposity on cognition by the β coefficient of 1 year of aging on cognition, thereby yielding a comparison metric for other exposures in relation to the effect of a 1 year of age increase on the cognitive scores. The population attributable risk (PAR)^21^ of each modifiable factor was calculated from logistic regression models. In these models, reduced cognition was defined as a DSST score less than 1 SD below the mean (based on CAHHM data) or a MoCA score less than 26. Modifiable exposures were categorized as follows: the top 3 quartiles of BF or VAT quartiles were compared to the lowest; the IHRS was categorized into low-, moderate-, or high-risk categories; and educational level was dichotomized into completed high school or less vs any further education. A 2-sided *P* < .05 was considered nominally significant with no adjustment for multiple testing. All analyses were completed using SAS software, version 9.4 (SAS Institute Inc), and plots were generated using R software, version 4.0.2 (R Foundation for Statistical Computing).

## Results

A total of 9189 adults (mean [SD] age, 57.8 [8.8] years; 5179 [56.4%] women; and 1013 [11.0%] East and Southeast Asian; 295 [3.2%] South Asian; 7702 [83.8%] White European; and 179 [1.9%] other, including Black, Indigenous, mixed, and unknown ethnicity) participated in the study (Table 1). The 9189 participants free of clinical CVD underwent a cardiovascular risk factor assessment and cognitive testing; 9166 underwent percentage of BF assessment, and 6773 underwent MRI of abdominal adipose tissue. The mean (SD) non–laboratory-based IHRS was 10.7 (5.9) and was higher in men (12.3 [6.0]) compared with women (9.5 [5.6]). As expected, women had higher percentage BF (35.6% [8.1%]) compared with men (25.1% [6.8%]), but men had higher mean VAT volume than women (83.6 vs 61.4 mL). On the basis of the WHR, 2679 men (66.8%) had central obesity (WHR >0.90) compared with 1911 women (36.9%) (WHR >0.85). The mean (SD) cognitive scores were 72.6 (16.0) on the DSST and 27.0 (2.4) on the MoCA, and both measures were higher in women (75.7 [15.7] on the DSST and 27.1 [2.3] on the MoCA) compared with men (68.6 [15.3] on the DSST and 26.7 [2.4] on the MoCA (Table 1). These differences remained significant when adjusted for age, ethnicity, and educational level.

**Table 1.** Baseline Characteristics Overall and by Sex^a^

Characteristic	Overall (N = 9189)	Women (n = 5179)	Men (n = 4010)
Age, mean (SD), y	57.8 (8.8)	57.4 (8.6)	58.3 (9.0)
Ethnicity			
East and Southeast Asian	1013 (11.0)	578 (11.2)	435 (10.8)
South Asian	295 (3.2)	129 (2.5)	166 (4.1)
White European	7702 (83.8)	4361 (84.2)	3341 (83.3)
Other^b^	179 (1.9)	111 (2.1)	68 (1.7)
Highest level of education completed (N = 9021)			
Primary, none, or unknown	94 (1.0)	44 (0.9)	50 (1.3)
High school	1469 (16.3)	927 (18.2)	542 (13.8)
Trade or vocational	873 (9.7)	423 (8.3)	450 (11.4)
College or university	6585 (73.0)	3687 (72.6)	2898 (73.6)
Cognitive function scores			
DSST, mean (SD)	72.6 (16.0)	75.7 (15.7)	68.6 (15.3)
MoCA, mean (SD)	27.0 (2.4)	27.1 (2.3)	26.7 (2.4)
Body fat, mean (SD), % (n = 9166)	31.0 (9.2)	35.6 (8.1)	25.1 (6.8)
MRI-detected visceral adipose tissue, mean (SD), mL (n = 6773)	71.2 (36.5)	61.4 (30.3)	83.6 (39.8)
MRI-detected vascular brain injury (n = 9116)	864 (9.5)	466 (9.1)	398 (10.0)
IHRS, mean (SD)			
Non–laboratory based	10.7 (5.9)	9.5 (5.6)	12.3 (6.0)
Laboratory based (n = 5349)	10.8 (5.8)	9.2 (5.4)	12.6 (5.8)
BMI, mean (SD) (n = 9184)	26.9 (4.9)	26.6 (5.4)	27.4 (4.1)
<25 (Normal)	3550 (38.6)	2325 (44.9)	1225 (30.6)
25-29 (Overweight)	3535 (38.5)	1658 (32.0)	1877 (46.8)
≥30 (Obese)	2100 (22.9)	1193 (23.0)	907 (22.6)
Waist to hip ratio (n = 9184)	0.87 (0.09)	0.83 (0.07)	0.93 (0.07)
Waist to hip ratio obesity (n = 9184)	4590 (50.0)	1911 (36.9)	2679 (66.8)
Self-reported history of diabetes	424 (4.6)	185 (3.6)	239 (6.0)
Hypertension	3553 (38.7)	1602 (30.9)	1951 (48.7)
Blood pressure, mean (SD), mm Hg (n = 9186)			
Systolic	129.3 (17.0)	125.5 (17.0)	134.2 (15.6)
Diastolic	79.8 (10.1)	78.1 (9.9)	81.9 (9.9)
Smoking status			
Current (in past year)	612 (6.7)	339 (6.5)	273 (6.8)
Former (quit >1 y ago)	3121 (34.0)	1710 (33.0)	1411 (35.2)
Never	5456 (59.4)	3130 (60.4)	2326 (58.0)
Second-hand smoke exposure (≥1 h per week)	394 (4.3)	225 (4.3)	169 (4.2)
Family history of myocardial infarction	3244 (35.3)	1934 (37.3)	1310 (32.7)

Abbreviations: BMI, body mass index (calculated as weight in kilograms divided by height in meters squared); DSST, Digital Symbol Substitution Test; IHRS, INTERHEART Risk Score; MoCA, Montreal Cognitive Assessment; MRI, magnetic resonance imaging.

^a^ Data are presented as number (percentage) of patients unless otherwise indicated.

^b^ Includes Black, Indigenous, mixed, and unknown ethnicity.

### Cardiovascular Risk Factors and Preclinical Vascular Disease

Higher total percentage of BF and VAT were each associated with changes in cardiovascular risk factors. There was an increasing trend across quartiles of BF percentage and VAT for hypertension (BF percentage quartile 4: 55.6%; 95% CI, 53.4%-57.8%; VAT quartile 4: 56.0%; 95% CI, 53.4-58.6), diabetes (BF percentage quartile 4: 8.3%; 95% CI, 7.2%-9.6%; VAT quartile 4: 9.0%; 95% CI, 7.7%-10.6%), and apolipoprotein B (BF percentage quartile 4: 1.05 g/L; 95% CI, 1.03-1.06 g/L; VAT quartile 4: 1.04 g/L; 95% CI, 1.02-1.05 g/L), and reduced apolipoprotein A1 (BF percentage quartile 4: 1.48 g/L; 95% CI, 1.47-1.50 g/L; VAT quartile 4: 1.45 g/L; 95% CI, 1.44-1.47 g/L) (Table 2).

**Table 2.** Risk Factors and Cognitive Function Scores by Adiposity Quartiles^a^

Variable	Bioimpedance total body adiposity					Visceral adipose tissue				
	Percentage of BF sex-specific quartiles				*P* value for trend^b^	Visceral adipose tissue sex-specific quartiles				*P* value for trend^b^
	1	2	3	4		1	2	3	4	
Quartile ranges										
Women	3.6-30.3	30.4-35.8	35.9-41.4	41.5-71.7	NA	7.6-<39.5	39.5-<54.6	54.6-<76.7	76.7-283.0	NA
Men	3.9-20.6	20.7-24.8	24.9-29.3	29.4-57.8		11.4-<54.3	54.3-<76.9	76.9-<105.5	105.5-303.2	
IHRS										
Score	8.3 (8.0-8.5)	9.7 (9.5-9.9)	11.2 (11.0-11.4)	13.8 (13.5-14.0)	<.001	7.3 (7.1-7.6)	8.7 (8.5-8.9)	10.3 (10.1-10.5)	13.3 (13.0-13.5)	<.001
Score without WHR	7.6 (7.4-7.8)	8.7 (8.5-8.9)	9.7 (9.5-9.9)	11.8 (11.6-12.0)	<.001	6.8 (6.5-7.0)	7.7 (7.5-8.0)	8.9 (8.7-9.1)	11.2 (10.9-11.4)	<.001
Risk score, laboratory based	7.7 (7.4-8.0)	9.4 (9.1-9.7)	11.1 (10.8-11.4)	13.6 (13.3-13.9)	<.001	6.6 (6.3-7.0)	8.5 (8.1-8.8)	10.3 (10.0-10.6)	13.3 (13.0-13.6)	<.001
Diabetes, %	1.7 (1.3-2.3)	2.4 (1.8-3.1)	4.3 (3.5-5.2)	8.3 (7.2-9.6)	<.001	1.3 (0.9-2.0)	1.8 (1.3-2.6)	3.7 (2.9-4.7)	9.0 (7.7-10.6)	<.001
Hypertension, %	22.1 (20.4-24.0)	31.5 (29.5-33.5)	40.0 (37.9-42.2)	55.6 (53.4-57.8)	<.001	20.0 (18.1-22.2)	27.1 (24.9-29.3)	39.1 (36.7-41.6)	56.0 (53.4-58.6)	<.001
Blood pressure, mm Hg										
Systolic	125 (124-125)	128 (127-128)	131 (130-131)	134 (133-135)	<.001	124 (123-125)	127 (126-128)	130 (129-131)	134 (133-134)	<.001
Diastolic	76 (76-76)	79 (78-79)	81 (80-81)	83 (83-84)	<.001	76 (75-76)	79 (78-79)	81 (80-81)	83 (82-83)	<.001
Waist to hip ratio										
Women	0.79 (0.78-0.79)	0.81 (0.81-0.82)	0.84 (0.84-0.84)	0.87 (0.87-0.87)	<.001	0.78 (0.78-0.79)	0.81 (0.81-0.81)	0.84 (0.84-0.84)	0.88 (0.88-0.89)	<.001
Men	0.88 (0.88-0.88)	0.92 (0.91-0.92)	0.94 (0.94-0.95)	0.98 (0.98-0.99)	<.001	0.87 (0.87-0.88)	0.91 (0.90-0.91)	0.94 (0.94-0.94)	0.99 (0.98-0.99)	<.001
Height, cm										
Women	161.4 (161.0-161.7)	162.6 (162.2-162.9)	162.6 (162.3-163.0)	163.0 (162.6-163.3)	<.001	162.0 (161.6-162.4)	162.7 (162.4-163.1)	162.8 (162.4-163.2)	162.8 (162.4-163.2)	.02
Men	176.2 (175.8-176.7)	175.7 (175.3-176.1)	175.6 (175.2-176.1)	175.0 (174.5-175.4)	<.001	175.1 (174.7-175.6)	175.7 (175.3-176.2)	175.4 (175.0-175.9)	176.3 (175.8-176.7)	.008
Apolipoprotein B, g/L	0.97 (0.95-0.98)	1.03 (1.01-1.04)	1.04 (1.03-1.05)	1.05 (1.03-1.06)	<.001	0.94 (0.93-0.96)	1.02 (1.01-1.04)	1.05 (1.04-1.07)	1.04 (1.02-1.05)	<.001
Apolipoprotein A1, g/L	1.63 (1.62-1.65)	1.59 (1.58-1.60)	1.53 (1.52-1.55)	1.48 (1.47-1.50)	<.001	1.64 (1.62-1.65)	1.58 (1.56-1.59)	1.52 (1.50-1.53)	1.45 (1.44-1.47)	<.001
Apolipoprotein B:A1 ratio	0.61 (0.60-0.62)	0.66 (0.65-0.67)	0.69 (0.68-0.71)	0.72 (0.71-0.73)	<.001	0.59 (0.58-0.60)	0.67 (0.65-0.68)	0.71 (0.70-0.73)	0.73 (0.72-0.74)	<.001
Brain magnetic resonance variables										
Silent brain infarction, %	3.3 (2.6-4.1)	3.4 (2.7-4.2)	4.1 (3.4-5.0)	4.6 (3.9-5.6)	.009	2.5 (1.8-3.3)	3.1 (2.4-4.1)	2.5 (1.8-3.3)	3.6 (2.8-4.5)	.16
Lacunar, %	2.3 (1.7-3.0)	2.2 (1.7-2.9)	3.0 (2.3-3.7)	3.6 (2.9-4.4)	.003	1.8 (1.2-2.6)	1.9 (1.3-2.6)	1.5 (1.1-2.2)	2.3 (1.7-3.2)	.43
Nonlacunar, %	1.0 (0.7-1.5)	1.1 (0.8-1.6)	1.1 (0.8-1.6)	1.1 (0.7-1.5)	.88	0.6 (0.3-1.1)	1.2 (0.8-1.8)	0.9 (0.5-1.4)	1.2 (0.8-1.8)	.17
Silent brain infarction count of ≥3, %	0.1 (0.0-100)	0.1 (0.0-100)	0.1 (0.0-100)	0.1 (0.0-100)	.59	0.0 (0.0-100)	0.1 (0.0-100)	0.1 (0.0-100)	0.1 (0.0-100)	.45
High white matter hyperintensities, %	3.4 (2.8-4.2)	2.9 (2.4-3.6)	3.1 (2.5-3.8)	4.0 (3.3-4.8)	.21	2.8 (2.1-3.7)	3.2 (2.5-4.1)	3.1 (2.4-3.9)	3.7 (3.0-4.7)	.11
Vascular brain injury, %	6.6 (5.7-7.8)	6.2 (5.4-7.3)	6.8 (5.9-7.9)	8.6 (7.5-9.8)	.007	5.3 (4.3-6.5)	6.1 (5.1-7.3)	5.5 (4.6-6.7)	7.2 (6.0-8.4)	.05
Abdominal magnetic resonance variables										
Subcutaneous adipose tissue volume, mL	NA	NA	NA	NA	NA	85.3 (83.2-87.4)	113.4 (111.4-115.5)	137.1 (135.0-139.2)	169.4 (167.2-171.6)	<.001
Visceral-subcutaneous adipose tissue ratio	NA	NA	NA	NA	NA	0.48 (0.47-0.50)	0.55 (0.53-0.56)	0.62 (0.61-0.63)	0.78 (0.77-0.79)	<.001
BF, %	21.5 (21.4-21.6)	28.7 (28.5-28.8)	33.5 (33.4-33.6)	40.5 (40.4-40.7)	<.001	23.8 (23.5-24.0)	28.4 (28.1-28.7)	32.3 (32.0-32.5)	37.5 (37.2-37.7)	<.001
Cognitive function scores										
DSST score	73.9 (73.3-74.4)	73.2 (72.7-73.7)	72.8 (72.2-73.3)	70.9 (70.4-71.5)	<.001	75.3 (74.6-75.9)	74.9 (74.3-75.6)	74.6 (73.9-75.2)	72.8 (72.1-73.4)	<.001
DSST 1 SD below mean, %^c^	10.5 (9.2-11.9)	13.3 (11.9-14.8)	13.2 (11.9-14.7)	15.5 (14.0-17.1)	<.001	8.6 (7.3-10.2)	9.0 (7.7-10.5)	10.9 (9.5-12.4)	12.1 (10.7-13.8)	<.001
MoCA score	27.1 (27.0-27.1)	27.0 (26.9-27.1)	27.0 (26.9-27.1)	26.8 (26.7-26.9)	.003	27.2 (27.1-27.3)	27.2 (27.1-27.3)	27.1 (27.0-27.2)	27.1 (27.0-27.2)	.19
MoCA score <26, %	20.8 (19.1-22.7)	22.1 (20.3-23.9)	20.4 (18.7-22.1)	24.1 (22.3-26.0)	.06	19.5 (17.5-21.6)	18.8 (17.0-20.8)	20.0 (18.1-22.0)	19.6 (17.7-21.6)	.76

Abbreviations: BF, body fat; DSST, Digital Symbol Substitution test; IHRS, INTERHEART Risk Score; MoCA, Montreal Cognitive Assessment; NA, not applicable; WHR, waist to hip ratio.

^a^ Data are presented as means or percentages (95% CIs) adjusted for age, sex, and ethnicity. Both DSST and MoCA are further adjusted for educational level. Apolipoprotein concentrations were available in a subset of 4492 participants. Diabetes data are from participants with any type (including type 1 and type 2 diabetes) and taking medication for diabetes. Hypertension data are from those taking blood pressure–reducing medication or with an elevated blood pressure reading as determined by systolic blood pressure greater than 140 mm Hg or diastolic blood pressure greater than 90 mm Hg.

^b^ *P* value for trend calculated using linear contrasts.

^c^ A DSST score 1 SD below mean is calculated based on the Canadian Alliance for Healthy Hearts and Minds participants data as a value less than 1 SD (15.7) below the mean (74.0).

The IHRS in quartile 4 of BF percentage was 13.8 (95% CI, 13.5-14.0; *P <* .001 for trend) and in quartile 4 of VAT was 13.3 (95% CI, 13.0-13.5; *P <* .001 for trend). Accordingly, the percentages of BF and VAT were each positively associated with central adiposity and the IHRS (*P* < .001 for trend). The percentage of BF was strongly correlated with VAT volume (*r* = 0.76 in women; *r* = 0.70 in men; *P* < .001).

### MRI-Detected Vascular Brain Injury

Higher total percentage of BF was associated with greater MRI-detected vascular brain injury (with the fourth quartile value at 8.6% [95% CI, 7.5-9.8]; *P* < .007 for trend), largely associated with higher HWMHs and lacunar infarctions. Similar associations were observed for higher VAT and greater MRI-detected vascular brain injury (with increasing VAT with the fourth quartile value at 7.2% [95% CI, 6.0%-8.4%]; *P* = .05 for trend) (Table 2).

### Adiposity and Cognitive Function

Higher total percentage of BF was associated with lower DSST (with the fourth quartile score of 70.9 [95% CI, 70.4-71.5]; *P* < .001 for trend) and MoCA (with the fourth quartile score of 26.8 [95% CI, 26.7-26.9]; *P* = .003 for trend) scores in models adjusted for age, sex, educational level, and race and ethnicity. Higher VAT was also associated with lower DSST scores (with the fourth quartile score of 72.8 [95% CI, 72.1-73.4]; *P* < .001 for trend) but not with MoCA scores (with the fourth quartile value of 27.1 [95% CI, 27.0-27.2]; *P* = .19 for trend) (Table 2).

#### Multivariable Prediction Models

**Figure 1.**
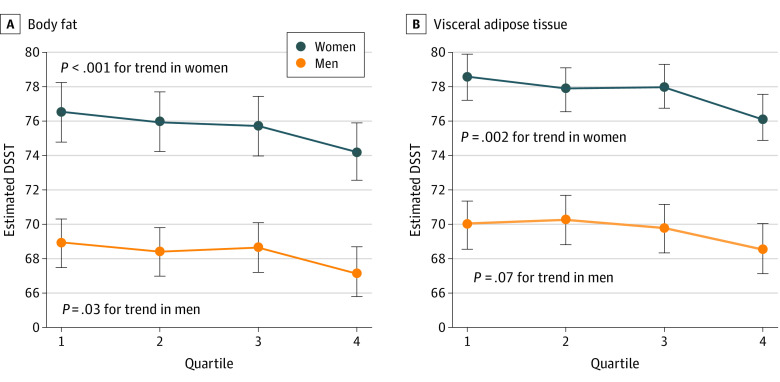
Body Fat and Visceral Adipose Tissue Association With Digital Symbol Substitution Test (DSST) Stratified by Sex A, The fully adjusted model shows lower cognitive scores measured by DSST with higher body fat percentage in men and women. B, The fully adjusted model shows lower cognitive scores measured by DSST with higher visceral adipose tissue in men and women.

In the maximally adjusted model that included age, sex, educational level, ethnicity, cardiovascular risk factors, and MRI-detected vascular brain injury, total percentage of BF remained independently associated with reduced cognitive scores. For each 1-SD increase in adiposity (corresponding to a 9.2% increase in BF or 36 mL of VAT), there was a reduction of 0.8 in the DSST cognitive score, which is equivalent to 1.0 year of cognitive aging. Being in the highest quartile vs the lowest quartile of sex-specific percentage of BF was associated with a 2.0 (95% CI, 1.1-2.8; *P* < .001) point lower DSST score (Figure 1; eTable 2 in the Supplement). This finding is equivalent to 2.8 years of cognitive aging. A similar magnitude of reduction in the DSST score of 2.0 (95% CI, 0.9-3.0; *P* < .001) was observed for VAT comparing the highest to the lowest quartile and is equivalent to 2.8 years of cognitive aging. In the sensitivity analyses, these associations remained of similar magnitude and significance in the healthy cohort subset (those participants without treated hypertension or diabetes) and when WHR was excluded from the IHRS (eTables 3 and 4 in the Supplement). Percentage of BF and VAT had no associations with the MoCA scores (reduced by 0.04; 95% CI, −0.10 to 0.02; *P* = .19).

**Figure 2.**
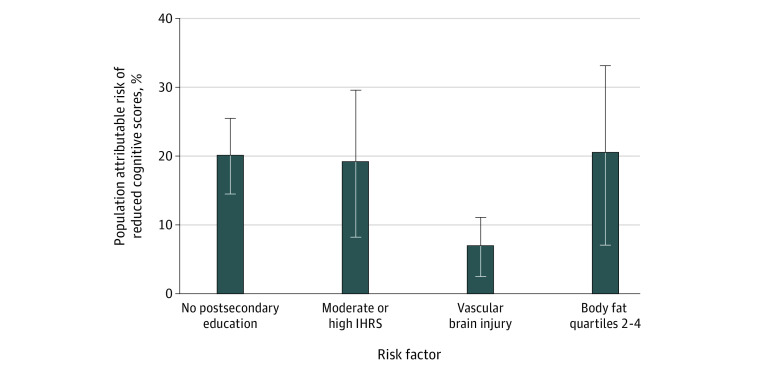
Population Attributable Risk (PAR) of Key Exposures on Reduced Cognitive Scores The greatest PAR on low Digital Symbol Substitution Test (DSST) score is the cardiovascular risk score, followed by adiposity, low educational level, and vascular injury. Error bars indicate 95% CIs. IHRS indicates INTERHEART Risk Score.

When the contributions of all factors included in the multivariable model were assessed on low cognitive scores defined as a DSST score less than 1 SD below the mean, the PAR of having high and moderate vs low IHRS was 19.2% (95% CI, 8.3-29.6), having less than high school education was 20.1% (95% CI, 14.5-25.5), having MRI vascular brain injury was 6.9% (95% CI, 2.6-11.1), and having higher percentage of BF in the top 3 quartiles vs the lowest was 20.5% (95% CI, 7.0%-33.2%) (Figure 2). Similar patterns and PARs were observed for VAT, with the PAR of having high and moderate vs low IHRS of 14.5% (95% CI, 2.5%-26.0%), having less than high school education of 13.6% (95% CI, 8.4%-18.8%), having MRI vascular brain injury of 5.7% (95% CI, 0.9%-10.5%), and having higher percentage of BF in the top 3 quartiles vs the lowest of 19.6% (95% CI, 2.0%-36.0%) (Table 3).

**Table 3.** Odds of Reduced Cognitive Function and PARs^a^

Variable	Odds of reduced cognitive function assessing percentage of BF quartiles (n = 8935)			Odds of reduced cognitive function assessing VAT quartiles (n = 6586)		
	Odds ratio (95% CI)	*P* value	Partial PAR (95% CI)	Odds ratio (95% CI)	*P* value	Partial PAR (95% CI)
Age (per 10 y)	2.72 (2.50-2.96)	<.001	NA	2.66 (2.39-2.96)	<.001	NA
Women	0.36 (0.30-0.43)	<.001	NA	0.32 (0.26-0.41)	<.001	NA
Height, cm	0.99 (0.98-1.00)	.005	NA	0.98 (0.97-1.00)	.009	NA
No postsecondary education	1.98 (1.71-2.28)	<.001	20.1 (14.5-25.5)	2.01 (1.65-2.44)	<.001	13.6 (8.4-18.8)
IHRS						
High vs low risk	1.37 (1.15-1.62)	<.001	19.2 (8.3-29.6)	1.26 (1.01-1.56)	.04	14.5 (2.5-26.0)
Moderate vs low risk	1.16 (1.00-1.35)	.05		1.18 (0.99-1.41)	.07	
Vascular brain injury	1.34 (1.12-1.60)	.001	6.9 (2.6-11.1)	1.35 (1.08-1.69)	.009	5.7 (0.9-10.5)
Percentage of BF				VAT quartiles		
Quartile 2 vs 1	1.31 (1.09-1.58)	.005	20.5 (7.0-33.2)	1.05 (0.83-1.34)	.68	19.6 (2.0-36.0)
Quartile 3 vs 1	1.21 (1.00-1.46)	.05		1.27 (1.00-1.61)	.05	
Quartile 4 vs 1	1.43 (1.18-1.73)	<.001		1.42 (1.11-1.81)	.006	

Abbreviations: BF, body fat; IHRS, INTERHEART Risk Score; NA, not applicable; PAR, population attributable risk; VAT, visceral adipose tissue.

^a^ Logistic regression further adjusted for ethnicity and recruiting center. Reduced cognitive function was defined as those with a Digital Symbol Substitution Test score less than 1 SD (15.7) below the mean (74.0) based on the Canadian Alliance for Healthy Hearts and Minds participants.

## Discussion

This cross-sectional study found that among adults with no prior history of clinical CVD, total percentage of BF and VAT are significantly associated with reduced cognitive scores, after adjustment for other cardiovascular risk factors, and MRI-detected vascular brain injury. For each 1-SD increase in adiposity (corresponding to 9.2% increase in BF or 36 mL of VAT), there was a reduction of 0.8 in the DSST cognitive score, which is equivalent to 1.0 year of cognitive aging. Compared with those in the lowest quartile, those in the highest quartile of adiposity using either metric had a commensurate 3 years of cognitive aging.

It is well documented that increased adiposity is associated with several cardiovascular risk factors,^1^ and separately, large-scale epidemiologic studies confirm that cardiovascular risk factors are associated with cognitive impairment.^22^ Adiposity may affect cognitive function through cardiovascular health yet may also have independent effects on cognitive function, such as inducing proinflammatory adipokines. It is logical therefore to investigate whether adiposity is associated with reduced cognitive scores, independent of cardiovascular risk factors. In our analysis, total adiposity and VAT were associated with higher cardiovascular risk factors, including blood pressure, the frequency of diabetes, apolipoprotein B/A ratio, and MRI-detected vascular brain injury. We also found that both adiposity metrics were associated with reduced cognitive scores, independent of cardiovascular risk factors and MRI-detected vascular brain injury.

The presence of VAT does not appear to confer greater risk over percentage of BF in its association with cognitive scores. This finding is likely because these measurements are highly correlated (*r *≥ 0.70 in women and men), and participants in the highest quartile of total body adiposity and VAT had a similar mean adjusted IHRS (13.8 vs 13.3). A prior study^11^ found that body mass index was not strongly associated with cognitive scores, whereas central adiposity was, highlighting the limitation of body mass index as a measure of adiposity because it is numerically higher with increases in lean and adipose tissue mass.

The association between greater adiposity and lower cognitive scores was also more pronounced with the DSST measure of processing speed than with the multidimensional cognitive test MoCA. High performance on the DSST requires intact processing speed, visual scanning, attention, and working memory. The association of adiposity with DSST was independent of MRI-detected vascular brain injury, cardiovascular score, and level of education—factors that have previously been shown to be strongly associated with reduced DSST scores.^16^ In contrast, the MoCA is a global cognitive screen designed to assess cognitive impairment across multiple domains, such as verbal comprehension and memory, in an elderly population. The lack of association of adiposity with MoCA may be because adiposity is less strongly associated with some cognitive domains included in the MoCA or may reflect less sensitivity of the MoCA to capture subtle changes in cognition. Future evaluations of excess adiposity with additional tests of cognitive function inclusive of verbal and performance IQ are needed to further understand the role of adiposity on cognitive function.^23^

The PAR analysis indicated that the factors that associated with the greatest amount of cognitive dysfunction included (1) cardiovascular risk factors summarized as the IHRS, which includes an indirect measure of adiposity (WHR); (2) directly measured adiposity, which had the greatest association with reduced cognitive scores; and (3) educational level, all of which had a substantially greater association on reduced cognitive scores than did the presence of MRI-detected vascular brain injury. Future investigations, including mechanistic studies and randomized clinical trials, are required to elucidate the pathways by which high levels of adiposity reduce cognitive scores, independent of its effect on other cardiovascular risk factors. Several large prospective studies have shown that mild systemic inflammation is associated with the risk of cognitive impairment in adolescents^24^ and adults^25^ and with the outcome of dementia.^22,26^ Furthermore, a recent analysis^27^ of 15 000 participants in the UK Biobank identified that the association between adiposity and reduced cognitive function was mediated through adipose tissue’s effects on traditional cardiovascular risk factors and inflammatory markers. Higher peripheral inflammation has also been observed to be associated with poorer spatial reasoning, short-term memory, verbal proficiency, learning and memory, and executive function, as well as structural changes in the brain, including lower cortical gray and white matter volumes, hippocampal volume, and cortical surface area.^28^ A recent genome-wide association study^29^ that identified inflammatory pathways associated with dementia also supports a link between inflammation and reduced cognitive function. Future studies that combine large-scale genomics with the detailed adiposity measures, brain phenotyping (ie, imaging genomics) and possible incorporation of novel study designs, such as mendelian randomization, may help elucidate unique causal pathways underpinning these associations.^30^

Cross-sectional or prospective observational studies are important contributions to this field because there have been a limited number of randomized clinical trials with precise measures of VAT or other ectopic adipose tissue depots and cognitive function. A small randomized clinical trial^31^ of weight loss in children with obesity showed that cognitive function scores improved among children when VAT was significantly lowered with weight loss. Other trials have evaluated the effect of reducing inflammation on cognitive function,^32^ depression, and dementia, and although promising, they are as yet inconclusive.^33^

### Strengths and Limitations

The strength of this study is that the findings are generalizable because they are derived from a robust cross-sectional analysis of healthy men and women, which suggests that the associations of adiposity measures with cognitive function persist after adjustment for established cardiovascular risk factors and educational level.

The limitation of this analysis is our inability to test for causality between increased adiposity and reduced cognitive function. Cross-sectional studies are at risk of reverse causation bias, although in this case because MRI detects subclinical measures of VAT and vascular brain injury and cognitive scores were only mildly reduced, it is unlikely that reverse causation played a significant role. Individuals with diabetes or hypertension may change their lifestyles after diagnosis of these conditions; therefore, we performed a sensitivity analysis to remove these participants from the analyses, which did not alter the association between adiposity and cognitive scores.

## Conclusions

This cross-sectional study found that excess adiposity was a risk factor for reduced cognitive scores, independent of cardiovascular risk factors, educational level, and MRI-detected vascular brain injury. Strategies to prevent or reduce adiposity may preserve cognitive function among adults.

## Notes

Supplement. **eTable 1.** STROBE Statement—Checklist of Items That Should Be Included in Reports of Cross-Sectional Studies **eTable 2.** Effect of Adiposity and Demographics on Cognition, as Measured by the DSST **eTable 3.** Healthy Cohort: Effect of Adiposity on Cognition, as Measured by the DSST **eTable 4.** Waist to Hip Ratio Removed From IHRS: Effect of Adiposity on Cognition, as Measured by the DSST **eFigure.** Consort Diagram
