# Supplementary material for: Evaluation of Adiposity and Cognitive Function in Adults
Source: JAMA Netw Open. 2022 Feb 1;5(2):e2146324. doi: 10.1001/jamanetworkopen.2021.46324 (PMC8808326; doi:10.1001/jamanetworkopen.2021.46324)
Supplement: Supplement. — eTable 1. STROBE Statement—Checklist of Items That Should Be Included in Reports of Cross-Sectional Studies eTable 2. Effect of Adiposity and Demographics on Cognition, as Measured by the DSST eTable 3. Healthy Cohort: Effect of Adiposity on Cognition, as Measured by the DSST eTable 4. Waist to Hip Ratio Removed From IHRS: Effect of Adiposity on Cognition, as Measured by the DSST eFigure. Consort Diagram [file jamanetwopen-e2146324-s001.pdf]

Supplemental Online Content

Anand SS, Friedrich MG, Lee DS, et al; Canadian Alliance of Healthy Hearts and Minds (CAHHM) and the Prospective Urban and Rural Epidemiological (PURE) Study Investigators. Evaluation of adiposity and cognitive function in adults. *JAMA Netw Open*. 2022;5(2):e2146324. doi:10.1001/jamanetworkopen.2021.46324

eTable 1. STROBE Statement—Checklist of Items That Should Be Included in Reports of Cross-Sectional Studies
eTable 2. Effect of Adiposity and Demographics on Cognition, as Measured by the DSST
eTable 3. Healthy Cohort: Effect of Adiposity on Cognition, as Measured by the DSST
eTable 4. Waist to Hip Ratio Removed From IHRS: Effect of Adiposity on Cognition, as Measured by the DSST
eFigure. Consort Diagram

This supplemental material has been provided by the authors to give readers additional information about their work.

© 2022 Anand SS et al. *JAMA Network Open.*

Supplementary e-Table 1: STROBE Statement—Checklist of items that should be included in reports of *cross-sectional studies*

	Item No	Recommendation	Page No
**Title and abstract**	1	(*a*) Indicate the study's design with a commonly used term in the title or the abstract	
		(*b*) Provide in the abstract an informative and balanced summary of what was done and what was found	2-3 (Abstract)
**Introduction**			
Background/rationale	2	Explain the scientific background and rationale for the investigation being reported	4 (Introduction)
Objectives	3	State specific objectives, including any prespecified hypotheses	4-5 (Introduction)
**Methods**			
Study design	4	Present key elements of study design early in the paper	5-6*(Methods)*
Setting	5	Describe the setting, locations, and relevant dates, including periods of recruitment, exposure, follow-up, and data collection	5 *(Methods)*
Participants	6	(*a*) Give the eligibility criteria, and the sources and methods of selection of participants	5, sFigure 1
Variables	7	Clearly define all outcomes, exposures, predictors, potential confounders, and effect modifiers. Give diagnostic criteria, if applicable	5-6 *(Methods, Statistical Analysis)*
Data sources/ measurement	8*	For each variable of interest, give sources of data and details of methods of assessment (measurement). Describe comparability of assessment methods if there is more than one group	*5-6 (Methods, Statistical Analysis)*
Bias	9	Describe any efforts to address potential sources of bias	7 (Methods, Statistical Analysis)
Study size	10	Explain how the study size was arrived at	sFigure1
Quantitative variables	11	Explain how quantitative variables were handled in the analyses. If applicable, describe which groupings were chosen and why	7 (Statistical Analysis)
Statistical methods	12	(*a*) Describe all statistical methods, including those used to control for confounding	7 (Statistical Analysis)
		(*b*) Describe any methods used to examine subgroups and interactions	7 (Statistical Analysis)
		(*c*) Explain how missing data were addressed	7 (Statistical analysis)
		(*d*) If applicable, describe analytical methods taking account of sampling strategy	7 (Statistical analysis)
		(*e*) Describe any sensitivity analyses	7 (Statistical analysis)
**Results**			

© 2022 Anand SS et al. *JAMA Network Open.*

	Item No	Recommendation	Page No
Participants	13*	(a) Report numbers of individuals at each stage of study—eg numbers potentially eligible, examined for eligibility, confirmed eligible, included in the study, completing follow-up, and analysed	sFigure1
		(b) Give reasons for non-participation at each stage	sFigure1
		(c) Consider use of a flow diagram	sFigure1
Descriptive data	14*	(a) Give characteristics of study participants (eg demographic, clinical, social) and information on exposures and potential confounders	Table 1
		(b) Indicate number of participants with missing data for each variable of interest	Tables present these.
Outcome data	15*	Report numbers of outcome events or summary measures	Page 8 (results)
Main results	16	(*a*) Give unadjusted estimates and, if applicable, confounder-adjusted estimates and their precision (eg, 95% confidence interval). Make clear which confounders were adjusted for and why they were included	Presented in all results and tables.
		(*b*) Report category boundaries when continuous variables were categorized	Tables 2-3
		(*c*) If relevant, consider translating estimates of relative risk into absolute risk for a meaningful time period	Pages 8-9 (Results)
Other analyses	17	Report other analyses done—eg analyses of subgroups and interactions, and sensitivity analyses	Page 9 (Results, "Multivariable prediction models"
**Discussion**			
Key results	18	Summarise key results with reference to study objectives	Page 9-10
Limitations	19	Discuss limitations of the study, taking into account sources of potential bias or imprecision. Discuss both direction and magnitude of any potential bias	Page 12
Interpretation	20	Give a cautious overall interpretation of results considering objectives, limitations, multiplicity of analyses, results from similar studies, and other relevant evidence	Page 12, Conclusion
Generalisability	21	Discuss the generalisability (external validity) of the study results	Page 12
**Other information**			
Funding	22	Give the source of funding and the role of the funders for the present study and, if applicable, for the original study on which the present article is based	Page 14-15.

*Give information separately for exposed and unexposed groups.

© 2022 Anand SS et al. *JAMA Network Open.*

Note: An Explanation and Elaboration article discusses each checklist item and gives methodological background and published examples of transparent reporting. The STROBE checklist is best used in conjunction with this article (freely available on the Web sites of PLoS Medicine at http://www.plosmedicine.org/, Annals of Internal Medicine at http://www.annals.org/, and Epidemiology at http://www.epidem.com/). Information on the STROBE Initiative is available at www.strobe-statement.org.

Supplementary e-Table 2: Effect of Adiposity and demographics on Cognition, as measured by the DSST

	Model 1 DSST with continuous BF%		Model 2 DSST with BF% quartiles		Model 3 DSST with continuous VAT		Model 4 DSST with VAT quartiles		
	Mean Score effect (95% CI)	P-value	Mean Score effect (95% CI)	P-value	Mean Score effect (95% CI)	P-value	Mean Score effect (95% CI)	P-value	
Age (per 10 years)	-7.1 (-7.4,-6.8)	<.0001	-7.1 (-7.5,-6.8)	<.0001	-6.9 (-7.2,-6.5)	<.0001	-6.9 (-7.3,-6.5)	<.0001	Age (per 10 years)
Women	8.4 (7.5,9.4)	<.0001	7.6 (6.8,8.4)	<.0001	7.8 (6.8,8.7)	<.0001	8.2 (7.2,9.1)	<.0001	Women
High School or less vs College/University	-5.0 (-5.7,-4.2)	<.0001	-5.0 (-5.7,-4.2)	<.0001	-4.6 (-5.6,-3.7)	<.0001	-4.6 (-5.6,-3.7)	<.0001	High School or less vs College/University
Trade/Vocational vs College/University	-4.7 (-5.7,-3.8)	<.0001	-4.7 (-5.6,-3.8)	<.0001	-3.4 (-4.5,-2.3)	<.0001	-3.5 (-4.5,-2.4)	<.0001	Trade or Vocational vs College/University
IHRS (per 5 unit change)	-0.8 (-1.1,-0.6)	<.0001	-0.8 (-1.1,-0.5)	<.0001	-0.6 (-1.0,-0.3)	<0.001	-0.7 (-1.0,-0.3)	<.0001	IHRS (per 5 unit change)
Vascular Brain Injury	-1.4 (-2.4,-0.5)	0.003	-1.4 (-2.4,-0.5)	0.003	-1.3 (-2.5,-0.2)	0.02	-1.3 (-2.5,-0.2)	0.03	Vascular Brain Injury
Height (cm)	0.1 (0.1,0.2)	<.0001	0.1 (0.1,0.2)	<.0001	0.1 (0.1,0.2)	<.0001	0.1 (0.1,0.2)	<.0001	Height (cm)
BF% (per 1 SD increase)	-0.8 (-1.1,-0.4)	<.0001			-0.8 (-1.2,-0.4)	<0.001			Visceral Adipose Tissue (per 1 SD increase)
BF% quartile 2 vs 1			-0.4 (-1.2,0.3)	0.25			-0.3 (-1.2,0.6)	0.57	VAT quartile 2 vs 1
BF% quartile 3 vs 1			-0.5 (-1.3,0.3)	0.19			-0.4 (-1.4,0.5)	0.35	VAT quartile 3 vs 1

© 2022 Anand SS et al. *JAMA Network Open.*

	Model 1<br>DSST with continuous BF%		Model 2<br>DSST with BF% quartiles		Model 3<br>DSST with continuous VAT		Model 4<br>DSST with VAT quartiles		
	**Mean Score effect (95% CI)**	**P-value**	**Mean Score effect (95% CI)**	**P-value**	**Mean Score effect (95% CI)**	**P-value**	**Mean Score effect (95% CI)**	**P-value**	
BF% quartile 4 vs 1			-2.0 (-2.8,-1.1)	<.0001			-2.0 (-3.0,-0.9)	<0.001	VAT quartile 4 vs 1

Models 1 and 2 assessing BF% exposure, N=8935; Mixed models adjusted for ethnicity (fixed) and recruiting centre (random intercepts). Column 1 defines co-variates and categories of BF%.

Models 3 and 4 assessing VAT exposure, N=6,586; Mixed models adjusted for ethnicity (fixed) and recruiting centre (random intercepts). Column 10 defines co-variates and categories of VAT.

Cut points for BF% quartiles are: 20.6, 24.8 and 29.3 for men; 30.3, 35.8 and 41.4 for women.
Cut points for VAT quartiles are: 54.3, 76.9 and 105.5 for men; 39.5, 54.6 and 76.7 for women.

© 2022 Anand SS et al. *JAMA Network Open.*

e-Table 3 Healthy Cohort: Effect of Adiposity on Cognition, as measured by the DSST

	Model 1 DSST with continuous BF%		Model 2 DSST with BF% quartiles		Model 3 DSST with continuous VAT		Model 4 DSST with VAT quartiles		
	**Mean Score effect (95% CI)**	**P-value**	**Mean Score effect (95% CI)**	**P-value**	**Mean Score effect (95% CI)**	**P-value**	**Mean Score effect (95% CI)**	**P-value**	
Age (per 10 years)	-7.0 (-7.4,-6.6)	<0.001	-7.0 (-7.4,-6.6)	<0.001	-6.7 (-7.2,-6.2)	<0.001	-6.7 (-7.2,-6.2)	<0.001	Age (per 10 years)
Women	8.5 (7.3,9.8)	<0.001	8.1 (7.0,9.1)	<0.001	8.1 (6.9,9.3)	<0.001	8.5 (7.2,9.7)	<0.001	Women
High School or less vs College/University	-5.2 (-6.3,-4.2)	<0.001	-5.2 (-6.2,-4.1)	<0.001	-4.9 (-6.2,-3.6)	<0.001	-4.9 (-6.2,-3.6)	<0.001	High School or less vs College/University
Trade/Vocational vs College/University	-4.4 (-5.7,-3.1)	<0.001	-4.4 (-5.7,-3.1)	<0.001	-3.1 (-4.5,-1.6)	<0.001	-3.1 (-4.6,-1.6)	<0.001	Trade or Vocational vs College/University
IHRS (per 5 unit change)	-1.2 (-1.7,-0.8)	<0.001	-1.2 (-1.6,-0.7)	<0.001	-0.9 (-1.5,-0.4)	<0.001	-0.9 (-1.4,-0.4)	<0.001	IHRS (per 5 unit change)
Vascular Brain Injury	-1.1 (-2.5,0.4)	0.16	-1.1 (-2.5,0.4)	0.15	-0.8 (-2.6,0.9)	0.34	-0.8 (-2.5,0.9)	0.37	Vascular Brain Injury
Height (cm)	0.1 (0.1,0.2)	<0.001	0.1 (0.1,0.2)	<0.001	0.1 (0.1,0.2)	<0.001	0.1 (0.1,0.2)	<0.001	Height (cm)
BF% (per 1 SD increase)	-0.5 (-0.9,0.0)	0.05			-0.6 (-1.2,-0.1)	0.03			Visceral Adipose Tissue (per 1 SD increase)
BF% quartile 2 vs 1			-0.3 (-1.2,0.6)	0.54			-0.4 (-1.5,0.7)	0.46	VAT quartile 2 vs 1
BF% quartile 3 vs 1			-0.5 (-1.5,0.5)	0.36			-0.3 (-1.4,0.9)	0.64	VAT quartile 3 vs 1
BF% quartile 4 vs 1			-1.8 (-2.9,-0.7)	0.002			-2.0 (-3.4,-0.7)	0.003	VAT quartile 4 vs 1

Models 1 and 2 assessing BF% exposure, N=5,386; Mixed models adjusted for ethnicity (fixed) and recruiting centre (random intercepts); Column 1 defines co-variates and categories of BF%.
Models 3 and 4 assessing VAT exposure, N=4,069; Mixed models adjusted for ethnicity (fixed) and recruiting centre (random intercepts). Column 10 defines co-variates and categories of VAT.

© 2022 Anand SS et al. *JAMA Network Open.*

e-Table 4 Waist to Hip Ratio Removed from IHRS: Effect of Adiposity on Cognition, as measured by the DSST

	Model 1 DSST with continuous BF%		Model 2 DSST with BF% quartiles		Model 3 DSST with continuous VAT		Model 4 DSST with VAT quartiles		
	Mean Score effect (95% CI)	P-value	Mean Score effect (95% CI)	P-value	Mean Score effect (95% CI)	P-value	Mean Score effect (95% CI)	P-value	
Age (per 10 years)	-7.1 (-7.5,-6.8)	<0.001	-7.1 (-7.5,-6.8)	<0.001	-6.9 (-7.3,-6.5)	<0.001	-6.9 (-7.3,-6.5)	<0.001	Age (per 10 years)
Women	8.9 (8.0,9.8)	<0.001	7.9 (7.1,8.7)	<0.001	7.9 (7.0,8.9)	<0.001	8.4 (7.5,9.4)	<0.001	Women
High School or less vs College/University	-5.0 (-5.8,-4.3)	<0.001	-5.0 (-5.8,-4.3)	<0.001	-4.7 (-5.6,-3.7)	<0.001	-4.7 (-5.6,-3.7)	<0.001	High School or less vs College/University
Trade/Vocational vs College/University	-4.8 (-5.7,-3.8)	<0.001	-4.7 (-5.7,-3.8)	<0.001	-3.4 (-4.5,-2.4)	<0.001	-3.5 (-4.6,-2.4)	<0.001	Trade or Vocational vs College/University
IHRS (per 5 unit change)	-0.8 (-1.1,-0.5)	<0.001	-0.8 (-1.1,-0.5)	<0.001	-0.6 (-0.9,-0.3)	<0.001	-0.6 (-0.9,-0.3)	<0.001	IHRS (per 5 unit change)
Vascular Brain Injury	-1.5 (-2.4,-0.5)	0.002	-1.5 (-2.4,-0.5)	0.002	-1.3 (-2.5,-0.2)	0.02	-1.3 (-2.5,-0.2)	0.02	Vascular Brain Injury
Height (cm)	0.1 (0.1,0.2)	<0.001	0.1 (0.1,0.2)	<0.001	0.1 (0.1,0.2)	<0.001	0.1 (0.1,0.2)	<0.001	Height (cm)
BF% (per 1 SD increase)	-0.9 (-1.2,-0.5)	<0.001			-0.9 (-1.2,-0.5)	<0.001			Visceral Adipose Tissue (per 1 SD increase)
BF% quartile 2 vs 1			-0.5 (-1.3,0.2)	0.18			-0.3 (-1.2,0.6)	0.49	VAT quartile 2 vs 1
BF% quartile 3 vs 1			-0.7 (-1.5,0.1)	0.09			-0.6 (-1.5,0.4)	0.23	VAT quartile 3 vs 1
BF% quartile 4 vs 1			-2.2 (-3.0,-1.4)	<0.001			-2.2 (-3.2,-1.2)	<0.001	VAT quartile 4 vs 1

Models 1 and 2 assessing BF% exposure, N=8,935; Mixed models adjusted for ethnicity (fixed) and recruiting centre (random intercepts); Column 1 defines co-variates and categories of BF%.
Models 3 and 4 assessing VAT exposure, N=6,586; Mixed models adjusted for ethnicity (fixed) and recruiting centre (random intercepts); Column 10 defines co-variates and categories of VAT.

© 2022 Anand SS et al. *JAMA Network Open*.

e-Figure 1: Consort Diagram

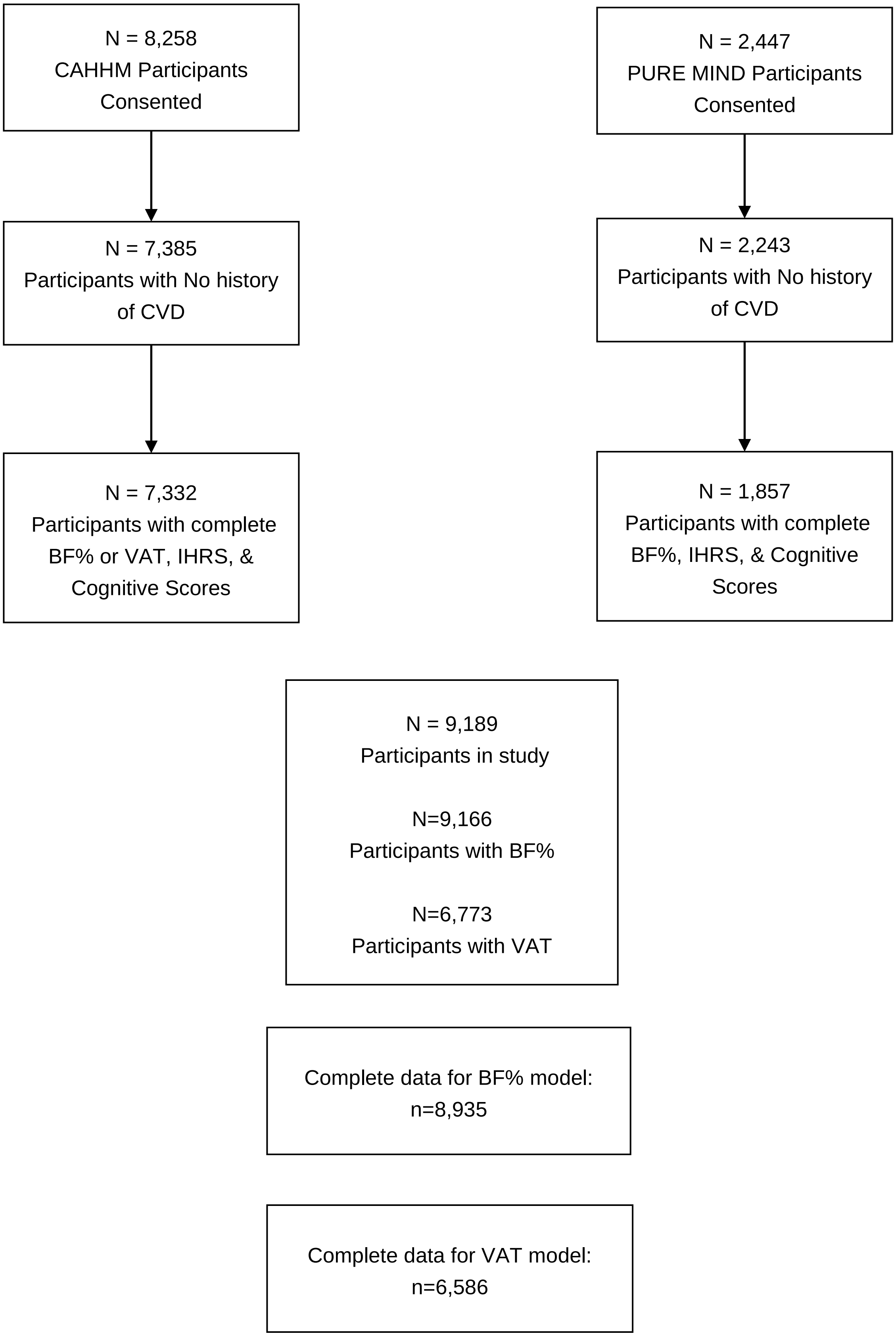

© 2022 Anand SS et al. *JAMA Network Open*.
